# SIV/SHIV-Zika co-infection does not alter disease pathogenesis in adult non-pregnant rhesus macaque model

**DOI:** 10.1371/journal.pntd.0006811

**Published:** 2018-10-25

**Authors:** Mehdi R. M. Bidokhti, Debashis Dutta, Lepakshe S. V. Madduri, Shawna M. Woollard, Robert Norgren, Luis Giavedoni, Siddappa N. Byrareddy

**Affiliations:** 1 Department of Pharmacology and Experimental Neuroscience, University of Nebraska Medical Center, Omaha, NE, United States of America; 2 Department of Genetics, Cell Biology and Anatomy, University of Nebraska Medical Center, Omaha, NE, United States of America; 3 Department of Virology and Immunology, Texas Biomedical Research Institute, San Antonio, TX, United States of America; 4 Department of Biochemistry and Molecular Biology, University of Nebraska Medical Center, Omaha, NE, United States of America; George Washington University, UNITED STATES

## Abstract

Due to the large geographical overlap of populations exposed to Zika virus (ZIKV) and human immunodeficiency virus (HIV), understanding the disease pathogenesis of co-infection is urgently needed. This warrants the development of an animal model for HIV-ZIKV co-infection. In this study, we used adult non-pregnant macaques that were chronically infected with simian immunodeficiency virus/chimeric simian human immunodeficiency virus (SIV/SHIV) and then inoculated with ZIKV. Plasma viral loads of both SIV/SHIV and ZIKV co-infected animals revealed no significant changes as compared to animals that were infected with ZIKV alone or as compared to SIV/SHIV infected animals prior to ZIKV inoculation. ZIKV tissue clearance of co-infected animals was similar to animals that were infected with ZIKV alone. Furthermore, in co-infected macaques, there was no statistically significant difference in plasma cytokines/chemokines levels as compared to prior to ZIKV inoculation. Collectively, these findings suggest that co-infection may not alter disease pathogenesis, thus warranting larger HIV-ZIKV epidemiological studies in order to validate these findings.

## Author summary

The co-infection incidence of human immunodeficiency virus (HIV) infection and neglected tropical infectious diseases such as Zika virus (ZIKV) is on the rise due to the large geographical overlap of populations exposed to both of these viruses. Thus, research on such co-infection is of particular importance. In this study, we investigated SIV/SHIV-ZIKV co-infection dynamics in adult non-pregnant Rhesus Macaques (RMs) chronically infected with simian immunodeficiency virus (SIV)—or chimeric simian human immunodeficiency virus (SHIV). We found that post ZIKV inoculation, ZIKV plasma viral loads in co-infected macaques were similar to ZIKV alone-infected animals, and minimal changes were observed in cytokines/chemokines levels. Viral levels of SIV and SHIV also did not change as compared to pre-ZIKV inoculation levels. These findings thus suggest that co-infection may not alter disease pathogenesis of either HIV or ZIKV infections.

## Introduction

Experimental and theoretical attention has been devoted to the interactions between human immunodeficiency virus (HIV) infection and various neglected tropical infectious diseases such as Zika virus (ZIKV), Dengue virus (DENV). These interactions could potentially lead to either pathogen altering the epidemiology, pathogenesis, immunology, and response to therapy of the other, sometimes even resulting in entirely new ailments that neither pathogen would have instigated alone [1]. In the last six decades since its discovery, Zika virus (ZIKV) has been considered a relatively mild human pathogen. Recently however, it has emerged as a threat to global health, demonstrating increased virulence, rapid spread and an association with grave neurological complications [2–4]. The two main types of clinical complications from ZIKV infection are microcephaly of newborns from women infected during early pregnancy [5], and a variety of neurological conditions in adults, including Guillain-Barré syndrome [1, 4] Serological tests cross-react DENV, and there are no specific antivirals or vaccines that are yet approved by Food and Drug Administration. Currently, the most effective tool for combating ZIKV is the prevention of mosquito bites, through measures such as repellents, protective nets, and insecticides [1].

The association between HIV infection and endemic diseases has been described in tropical regions with varying levels of complications. The first case of HIV-ZIKV co-infection was reported in Brazil without major health complications [6]. However, as the geographical range of ZIKV infection expands, exposed HIV immunosuppressed individuals may unveil new and more severe clinical manifestations, which must be anticipated. To the end, close surveillance of HIV-positive individuals to mirror such co-infections is of particular importance [1]. In this study, we investigated SIV/SHIV-ZIKV co-infection dynamics in a biologically relevant nonhuman adult non-pregnant primate model, with the objective of determining if and how ZIKV infection in HIV positive individuals may result in any potentially altered pathogenesis.

## Methods

### Animals and study design

**Fig 1:**
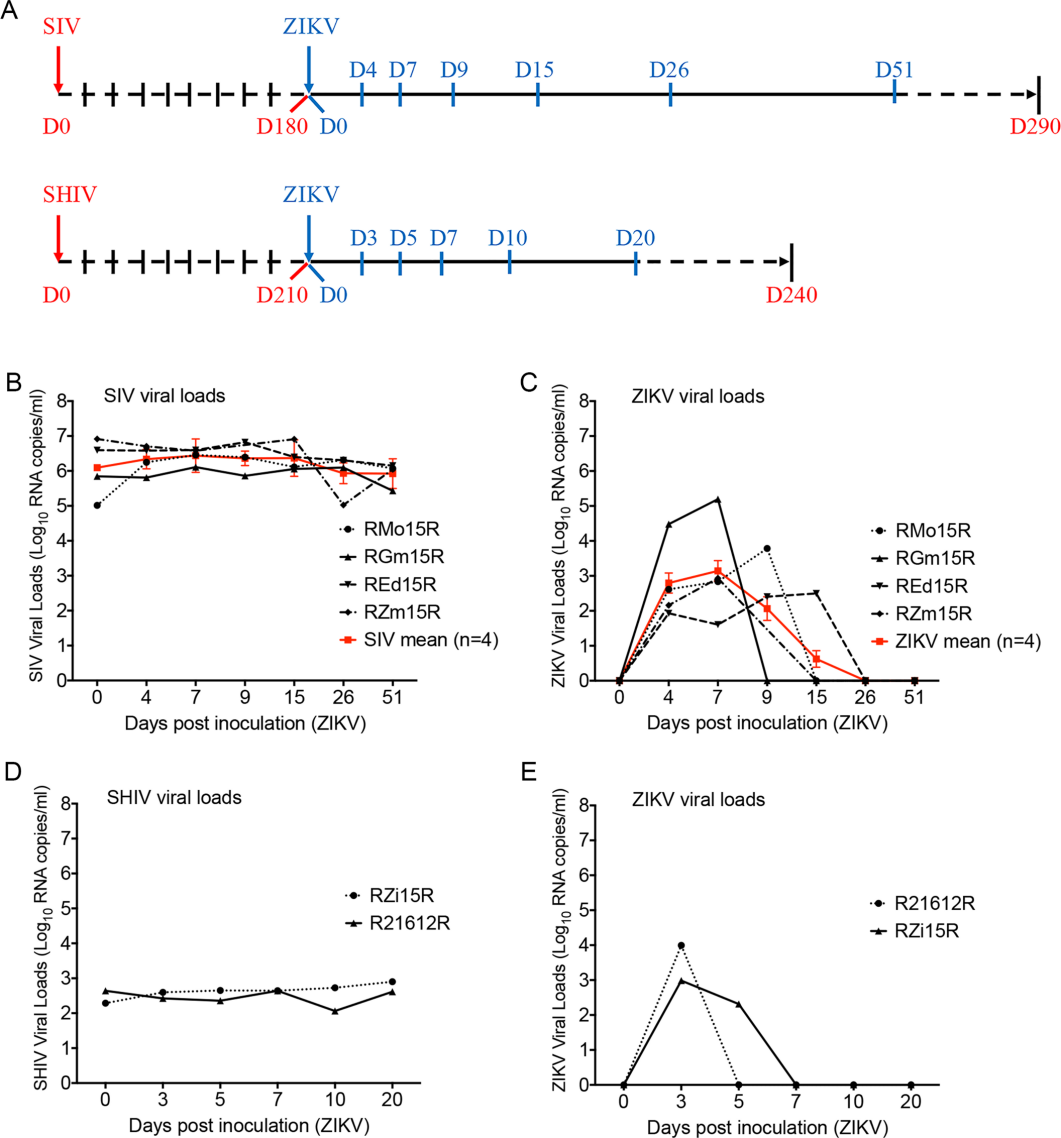
Viral loads of simian immunodeficiency virus, chimeric simian human immunodeficiency virus (SIV/SHIV) and Zika virus (ZIKV) in co-infected macaques. Female rhesus macaques (n = 6) chronically infected with SIVmac239 (n = 4) or SHIV3618MTF (n = 2) were also inoculated subcutaneously with 10^4^ plaque forming unit (PFU) of ZIKV PRVABC59. Blood collection was performed according to the study plan on 0, 4, 7, 9, 15, 26 and 51 days post inoculation (dpi) with ZIKV for SIV co-infected individuals and on 0, 3, 5, 7, 10 and 20 dpi with ZIKV for SHIV co-infected individuals. Day 0 (D0) was the day of inoculation with ZIKV. RNA was extracted from collected plasma samples through the use of QiAmp RNA mini kit (Qiagen, Valencia, CA), and viral loads were measured using one-step real time RT-PCR detection method. Viral loads were presented in Log10 RNA copies per milliliter (ml) of plasma. **A**, schema of time-course sampling in study plan of SIV (n = 4) and SHIV (n = 2) co-infection with ZIKV in rhesus macaques. **B**, viral load status of SIV in individual (black) and the mean value (red) of all SIV-ZIKV co-infected animals (n = 4) in days post ZIKV inoculation. **C**, viral load status of ZIKV in individual (black) and the mean value (blue) of all SIV-ZIKV co-infected animals (n = 4) in days post ZIKV inoculation. Bars indicate standard deviation (±SD) of mean values (n = 4). **D**, viral load status of SHIV in individual SHIV-ZIKV co-infected animals (n = 2) in days post ZIKV inoculation. **E**, viral load status of ZIKV in individual SHIV-ZIKV co-infected animals (n = 2) in days post ZIKV inoculation.

As described in **Fig 1A**, total of 6 adult female Indian-origin rhesus macaque (*Macaca mulatta;* age range 4.5 to 5 yrs) were chronically infected with SIVmac239 (n = 4) or SHIV3618MTF (n = 2) (newly developed clade C, T/F SHIV) [7] over a period of 6–7 months **(Table 1)**. These animals were inoculated subcutaneously with 10^4^ plaque forming unit (PFU) of ZIKV strain PRVABC59 **(Table 1)** and monitored for post ZIKV infection by viral loads and also evaluated for any clinical manifestation caused by ZIKV. All animal studies were conducted in accordance with UNMC IACUC approved protocols. Animal maintenance and procedures were carried out at the Department of Comparative Medicine, University of Nebraska Medical Center (UNMC) in accordance with the rules and regulations of the Committee on the Care and Use of Laboratory Animal Resources”. All protocols and procedures were performed under approval of the UNMC Institutional Animal Care and Use Committee according to the National Institute of Health guidelines.

**Table 1:** Details of the macaques utilized in this study.

Name of animals	Species of rhesus macaques	Type of inoculated SIV/SHIV	Type of inoculated ZIKV isolate	Gender	Date of birth	Date and route of SIV or SHIV inoculation	Weight on date of SIV or SHIV infection	Date and route of ZIKV inoculation	Date of Necropsy
RMo15R	Mulatta	SIVmac239	ZIKVPRVABC59	Female	6/25/2012	3/29/2016,IV^a^	5.54kg	9/26/2016, SC^c^	4/20/2017
RGm15R	Mulatta	SIVmac239	ZIKVPRVABC59	Female	5/30/2012	3/29/2016,IV	5.52kg	9/26/2016, SC	4/20/2017
REd15R	Mulatta	SIVmac239	ZIKVPRVABC59	Female	6/12/2012	3/29/2016,IV	6.12kg	9/26/2016, SC	3/21/2017
RZm15R	Mulatta	SIVmac239	ZIKVPRVABC59	Female	6/7/2012	3/29/2016,IV	5.46kg	9/26/2016, SC	1/17/2017
R21612R	Mulatta	SHIV3618MTF	ZIKVPRVABC59	Female	7/30/2012	9/20/2016, IVAG^b^	5.54kg	4/21/2017, SC	5/10/2017
RZi15R	Mulatta	SHIV3618MTF	ZIKVPRVABC59	Female	5/11/2012	9/20/2016, IVAG	5.56kg	4/21/2017, SC	5/10/2017

^a^ Intravenously.

^b^ Intravaginally.

^c^ Subcutaneously.

#### Sample collection and viral loads in plasma and tissues

On various days post inoculation (dpi) with ZIKV as depicted in (**Fig 1A)**, blood samples were collected and centrifuged to obtain plasma. Necropsy was performed 6–7 months post ZIKV infection. After perfusion with PBS, various organs were harvested and snap frozen for RNA extraction. To measure viral loads, RNA was extracted from plasma using QiAmp Viral RNA Mini Kit (Qiagen, CA). Quantitation of viral RNA was performed using the Taqman RNA-to-Ct 1-Step Kit (Thermo Fisher Scientific, MA), SIV and ZIKV viral loads were measured as described previously **[8, 9]**. Similarly, to measure ZIKV viral loads from tissues, RNA was extracted from various tissues and subjected to sensitive QX200 Droplet Digital PCR assay with limit of detection as low as 3 copies/ml (Bio-Rad, CA). A reaction mixture containing 200 ng of RNA was used in the One-Step RT-ddPCR advanced Kit along with primers and probes (Bio-Rad, Hercules CA). Micro droplets were generated using the QX200 Automated Droplet Generator (Bio-Rad, Hercules CA). Plates were sealed with the PX1 PCR Plate Sealer (Bio-Rad, Hercules CA) prior to PCR. Target cDNA was amplified with the C1000 Touch Thermal Cycler (Bio-Rad, Hercules CA) using the following conditions: 1 cycle 48°C for 1 hour, 1 cycle 95°C for 10 min, 40 cycles 95°C for 30 secs and 60°C for 1 min, 1 cycle 98°C for 10 min. After amplification, the plate was read on a QX200 Droplet Reader (Bio-Rad, Hercules CA) to determine the number of PCR-positive droplets vs. PCR-negative droplets in the original sample. Data acquisition and quantification was performed using QuantaSoft Software (Bio-Rad, Hercules CA). Furthermore, ZIKV viral loads from the plasma of SIV-ZIKV co-infected animals were compared with those of animals from a previous study, who were infected with ZIKV alone [10, 14].

#### Levels of cytokines and chemokines

**Fig 2:**
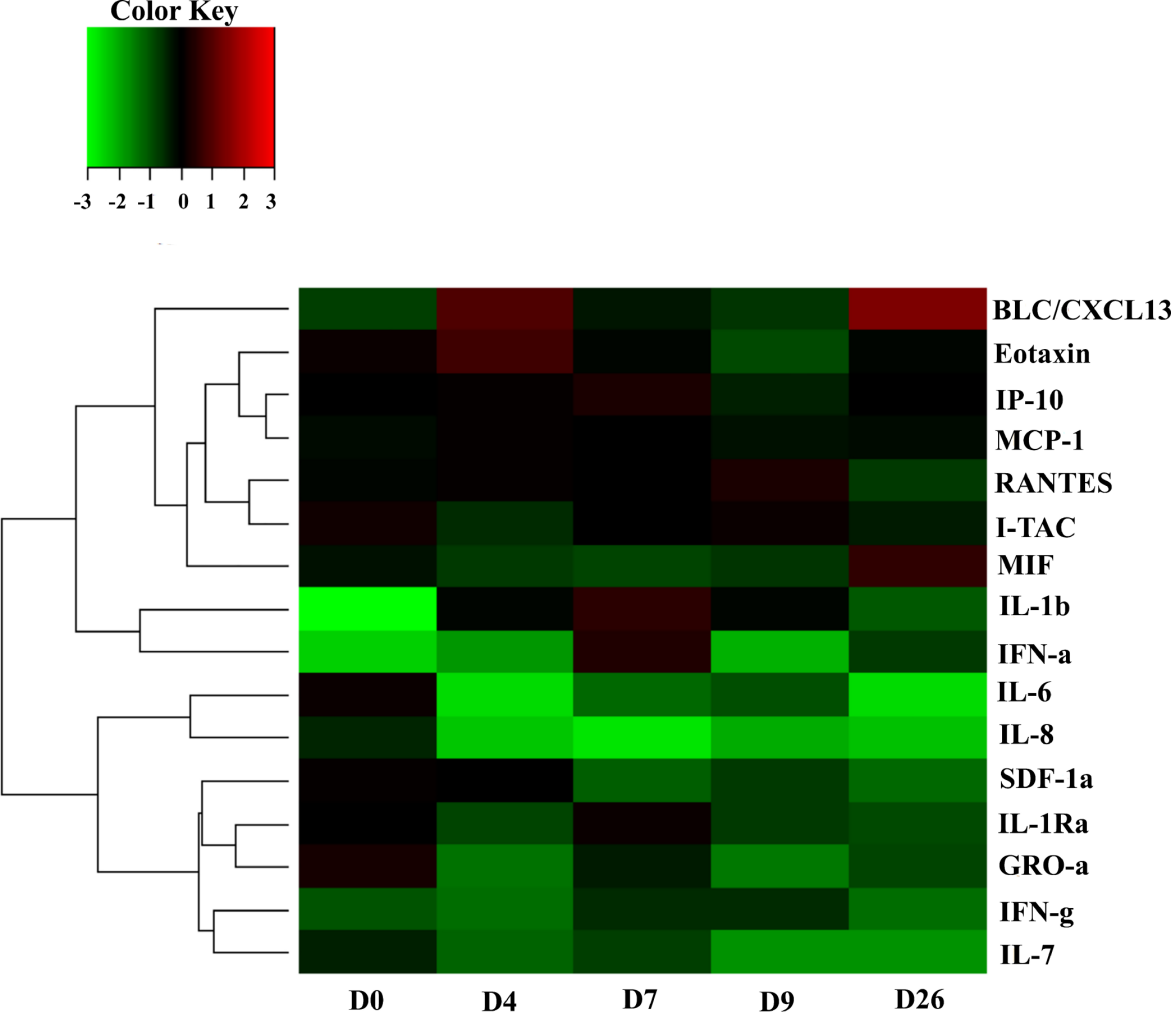
The heat map of cytokines/chemokines measurement screening in simian immunodeficiency virus—Zika virus (SIV-ZIKV) co-infected monkeys. Using plasma samples as described in Fig 1, D0 to D26 post inoculation with ZIKV, Luminex assay was performed to screen all relevant cytokines/chemokines measurements. Using R, a heat map was designed and drawn based on the median value (n = 3) of cytokines/chemokines measurement on D0, D4, D7, D9, and D26 post inoculation with ZIKV in SIV-ZIKV co-infected animals. Raw values for each of the cytokines/chemokines were normalized to the mean of the baselines (**S1A Table**), log2-transformed (**S1B Table**) and then subjected to hierarchical clustering. Heat maps were generated using heatmap 2 of R package plots. Values denoted as −3 to +3 represent a decrease to an increase in the levels relative to baseline values for each of chemokines/cytokines analyzed, that is, BLC, B lymphocyte chemoattractant; IP-10, interferon-γ-induced protein 10; MCP-1, monocyte chemoattractant protein-1; RANTES, regulated on activation, normal T-cell expressed and secreted; I-TAC, interferon-inducible T-cell alpha chemoattractant; MIF, macrophage migration inhibitory factor; IFN-α and -γ, interferon-α and -γ; IL, interleukin; SDF-1α, and stromal cell-derived factor.

Plasma samples from SIV-ZIKV co-infected macaques were analyzed through a commercially available Luminex methodology [11] for measuring the levels of cytokines and chemokines including: B lymphocyte chemo attractant (BLC)/CXCL13, Eotaxin, interferon-γ-induced protein 10 (IP-10), monocyte chemo attractant protein-1 (MCP-1), regulated on activation, normal T-cell expressed and secreted (RANTES), interferon-inducible T-cell alpha chemoattractant (I-TAC), macrophage migration inhibitory factor (MIF), interferon (IFN)-α, IFN-γ, interleukin (IL)-1β, IL-6, IL-7, IL-8, stromal cell-derived factor (SDF)-1α, IL-1Rα, and GRO-α, **(Fig 2, S1 Table)**.

#### Statistical analysis

ANOVA single, two-way without replication and two-way with replication was used to compare various data presented in this study.

## Results

### SIV/SHIV and Zika plasma/tissue viral load measurements

The SIV plasma viral loads of all the chronically infected SIV macaques were stable (10^5^−10^7^ copies/ml) and did not change even after ZIKV inoculation **(Fig 1B**). Additionally, ZIKV plasma viral loads were found to peak at 10^5^ copies/ml on 7 dpi **(Fig 1C**). ZIKV was detected in plasma samples up to 9 dpi in two of SIV-ZIKV co-infected animals; < 10^4^ copies/ml for RMo15R and > 10^2^ copies/ml for REd15R on 9 dpi. RGm15R that had the highest viral load, > 10^5^ copies/ml on 7 dpi, and was found negative to ZIKV on 9 dpi **(Fig 1C**). Interestingly, although REd15R had the lowest peak of ZIKV viral load of > 10^2^ copies/ml, ZIKV was still detected up to 15 dpi **(Fig 1C**). Furthermore, the SIV viral load status of these four SIV-ZIKV co-infected RMs was similar and quite stable during 51 dpi with ZIKV **(Fig 1B**).

In SHIV-ZIKV co-infected RMs, SHIV plasma viral loads were also found to be stable at 10^2^−10^3^ copies/ml during 20 dpi with ZIKV **(Fig 1D**). Additionally, ZIKV plasma viral loads were found to peak on 3 dpi to 10^3^ copies/ml for R21612R and 10^4^ copies/ml for RZi15R **(Fig 1E**). On 5 dpi, ZIKV was only detected at lower levels, < 10^3^ copies/ml, in RZi15R. Following this, ZIKV was never again detected in either RMs R21612R or RZi15R **(Fig 1E**).

The viral loads of ZIKV in all six RMs were found to be negative after 20 days onwards. However, this delay of self-recovery of viremia appeared to be longer in co-infected animals as compared to animals that were infected with ZIKV alone (**S1 and S2 Figs)**. However, the statistical analysis of ANOVA single (p value = 0.48), two-way without replication (p value = 0.42) and two-way with replication (p value = 0.51) of viral load of ZIKV did not reveal any significant differences between co-infected and exclusively ZIKV-infected RMs inoculated with 10^4^ PFU of ZIKV PRVABC59. Thus, the low sample size of our study does not provide enough evidence to confirm the significance of our observed delay of self-recovery from ZIKV viremia (**S1B Fig**), which may require a larger group of animals in future studies. Clinical investigation of co-infected RMs did not reveal any severe symptoms and/or clinical signs of ZIKV infection with permanent sequela after acute phase **(≤ 9 dpi)**. Additionally, at necropsy 6–7 months post-inoculation with ZIKV, various tissues including the brain stem, hippocampus, caudate, cerebellum, frontal cortex, spleen, mesenteric lymph node, uterus, liver, lung, and kidney, were collected and tested for ZIKV detection using a highly sensitive Droplet Digital PCR (ddPCR). We found that no detectable ZIKV viral RNA was present in any of the sampled tissues of three SIV-ZIKV co-infected RMs (RMo15R, RGm15R, and REd15R) and two of the SHIV infected RMs, suggesting that ZIKV infection had been cleared in these animals. This finding indicates the clearance of the ZIKV infection from the SIV-ZIKV co-infected adult RMs as similar to previously reported studies on RM that were infected with ZIKV alone [12, 13, 14].

### Measurement of cytokines/chemokines

Using Luminex methodology [11], cytokines and chemokines were measured from the plasma samples of three SIV-ZIKV co-infected RMs (RMo15R, RGm15R, and REd15R) on 0, 4, 7, 9, and 26 dpi with ZIKV. The results reveal that with the exception of MIF, IL-8, and SDF-1α, significant elevations of the plasma interleukin concentrations are evident in the acute phase of ZIKV especially at the peak on 7 dpi. Many of the cytokines and chemokines that were elevated in the acute phase of ZIKV displayed a tendency to return to normal levels in the later recovery phase (26 dpi) of ZIKV. Others however, such as BLC/CXCL13, Eotaxin, IP-10, MCP-1, and IFN-α, remained elevated during the peak of viremia (D4-D7) and also in recovery phase (D26). RANTES, I-TAC and, at lower levels, IL-1β, IL-6, IL-7, IFN-γ, SDF-1α, IL-1Rα, and GRO-α were elevated during acute phase and suppressed in recovery phase. Elevation of IL-1β and IFN-α in acute phase was noted. MIF and, at lower levels, IL-8 were suppressed during acute phase and elevated in recovery phase. The changes, either in the acute or in the recovery phase, were minor for MCP-1, IL-5, and IL-7 **(Fig 2, S1 Table)**.

## Discussion

The main objective of this study was to examine the dynamics of HIV-ZIKV co-infection in order to evaluate how one pathogen may affect the pathogenesis of the other. We used rhesus macaques that were chronically infected with either SIVmac239 or SHIV3618MTF. These macaques were later inoculated with 10^4^ PFU of ZIKV (PRVABC59) subcutaneously. In SIV-ZIKV co-infected RMs, Zika viral loads in plasma were found to be very similar to ZIKV infected animals drawn from both literature [10], and our own data [14]. Plasma viral loads of SIV and SHIV did not change as compared prior to ZIKV inoculation. These levels of viral load status of SIV and SHIV were also found to be very similar to chronically infected RMs drawn from both literature and our own data [15, 16].

Generally, mosquito-borne flaviviruses are initially detected in blood and lymphoid tissue of infected animals and subsequently invade peripheral organs and the central nervous system via the hematogenous route [2, 10]. In chronically infected SIV/SHIV RMs, ZIKV (PRVABC59) exhibited a similar pattern of viral kinetics as previously described in the ZIKV infected animals [10, 13]. The SIV/SHIV viremia kinetics in co-infected RMs were similar to those in the SIV/SHIV infected RMs from prior literature as well [15, 16]. Importantly, the rapid control of acute viremia of ZIKV infection that was observed in SIV/SHIV chronically infected RMs suggests that their peripheral immune system protects the host from peripheral ZIKV infection and that chronically infected SIV/SHIV RMs are similarly protected as non-immunocompromised RMs from infection by ZIKV.

All necropsies were performed 6–7 months after ZIKV infection, and ZIKV viral loads were measured in various tissues and organs using a highly sensitive Droplet Digital PCR and noted undetectable ZIKV viral loads. Prior studies had also revealed similar levels of clearance from tissue organs and body fluids for ZIKV (PRVABC59) infection in both humans [12] and RMs [13,14]. Human and animal model studies have demonstrated that ZIKV infection can result in persistence of infectious virus and viral nucleic acid in several body fluids (e.g., semen, saliva, tears, and urine) and target organs, including immune-privileged sites (e.g., eyes, brain, and testes) and the female genital tract [13, 17]. The ZIKV persistent or occult neurologic and lymphoid disease may occur following clearance of peripheral virus in ZIKV-infected individuals [12]. It has previously been demonstrated in infected RMs that ZIKV can persist in cerebrospinal fluid and lymph nodes for weeks after the virus has been cleared from peripheral blood, urine, and mucosal secretions [13]. The present adult RMs model confirmed that ZIKV persistent infection would also be cleared in immunocompromised RMs chronically infected with SIV. However, in this study we were unable to document the rate of any ZIKV clearance that occurred earlier than 6–7 months. ZIKV infection of rhesus and cynomolgus monkeys has been shown to recapitulate many key clinical findings, including rapid control of acute viremia, early invasion of the central nervous system, and prolonged viral shedding in adult animals [10, 13, 17]. Collectively, these findings enumerate that ZIKV infection in HIV infected individuals would not cause significant changes in pathogenesis and treatment plans.

Furthermore, besides the exceptions of MIF, IL-8, and SDF-1α, the cytokine/chemokine patterns of our RM were elevated during acute phase of ZIKV infection. Interestingly, this pattern is in accordance with previously described clinical findings for ZIKV-infected individuals [18, 19]. Major elevation was observed for IL-1β and IFN-α in acute phase ZIKV infection was observed in SIV-ZIKV co-infected RMs. The cytokine IL-1β is a key mediator of the inflammatory response and is essential for both host-response and resistance to pathogens. IFN-α is also mainly involved in innate immune responses against viral infection in acute phase of infection [11]. Together, these data confirm that ZIKV replication in the acute phase triggered rapid innate immune responses in peripheral blood. Several other chemoattractant chemokines (BLC/CXCL13, IP-10, MCP-1, RANTES, I-TAC, SDF-1α, and GRO-α) were also elevated during the ZIKV infection in chronically SIV/SHIV infected RMs described in this study, which is in accordance with previously described findings for ZIKV-infected individuals [19].

In this study IP-10 (CXCL10), has shown to be involved in ZIKV-related fetal neuron apoptosis or Guillain-Barré syndrome, and is suggested as potential biomarker of acute infection [19]. Specifically, these chemokines induce protective immunity against various viral infections including influenza, herpes simplex virus, Coxsackie virus, respiratory syncytial virus, and flaviviruses such as Dengue and West Nile viruses [18]. Elucidating the function of these chemokines in ZIKV infection, such as trafficking of lymphocytes into the various tissues, may reveal new mechanisms of immunological protection or immunopathology in SIV/SHIV-ZIKV co-infection. Additionally, although both B cell and T-cell recruitment to the sites of infection were highly triggered in SIV-ZIKV co-infected RMs in order to quickly control the infection, there were no significant changes between any of the cytokine levels measured in the acute versus the recovery phase.

Recent studies have revealed a significant increase in the number of HIV-ZIKV co-infected individuals reported in endemic areas in America [20–23] and Africa [24]. Additionally, the National Institute of Health (NIH) initiated an international, multisite clinical study, “Prospective Cohort Study of HIV and ZIKV in Infants and Pregnancy” (HIV ZIP; ClinicalTrials.gov, # NCT03263195) to register 2,000 pregnant women for the purpose of investigating ZIKV/HIV co-infection in the United States, Brazil, and Puerto Rico. Further studies suggest that although a high prevalence (72%) of ZIKV among 219 HIV-infected pregnant women was reported [20, 23]; their clinical presentation suggested a mild disease with a rapid and complete recovery. However, the fetuses of these women were often born with significant abnormalities, similar to those described previously in the children of women without HIV infection [25] and fetal demise would often occur [20]. In our current study, the cytokines/chemokines profile pattern following ZIKV infection SIV/SHIV models revealed a high level of similarity to the one described for HIV infected individuals [19]. Additionally, the notable similarity of ZIKV viral load pattern and clearance of ZIKV between SIV/SHIV models in this study and HIV infected individuals reported in these recent studies [20–25] highlights the utility of this co-infection model to understand disease pathogenesis.

In summary, we demonstrated that ZIKV viremia pattern in chronically infected SIV/SHIV RMs did not change significantly when compared to RMs that were infected with ZIKV alone, as well as to recent human epidemiological studies of HIV-ZIKV co-infection. These data suggest that ZIKV infection in chronically infected HIV individuals may not significantly alter the pathogenesis and disease progression of HIV or ZIKV, thus warranting larger epidemiological studies to validate these findings.

## Supporting information

S1 Fig Comparison of viral loads in simian immunodeficiency virus and chimeric simian human immunodeficiency virus—Zika virus (SIV/SHIV-ZIKV) co-infected macaques versus macaques infected with ZIKV alone. Rhesus macaques (RM; n = 6) chronically infected with either SIVmac239 (n = 4) or SHIV3618MTF (n = 2) were also inoculated subcutaneously with 10^4^ plaque forming unit (PFU) of ZIKV PRVABC59. The blood collections were performed according to the study plan on 0, 4, 7, 9, 15, 26 and 51 days post inoculation (DPI) with ZIKV for SIV co-infected RM and on 0, 3, 5, 7, 10, and 20 DPI with ZIKV for SHIV co-infected RM. Day 0 (D0) was the day of inoculation with ZIKV. RNA was extracted from collected plasma samples using the QiAmp RNA mini kit (Qiagen, Valencia, CA), and viral loads were measured using one-step real time RT-PCR detection method targeting Gag gene of SIV. Viral loads were presented in Log10 RNA copies per milliliter (ml) of plasma. **A)** Viral load status of ZIKV in SIV (black, this study), SHIV (orange, this study)–ZIKV co-infected RM, in ZIKV PRVABC59 (red)—infected RM, in ZIKV FrenchPolynesia, 2013 (brown)—infected RM and in ZIKV MR766 (blue)—infected RM in days post ZIKV inoculation. The reference number for animals studied previously is also given in their labels (https://zika.labkey.com/project/OConnor/begin.view); 295022 (infected with 10^4^ PFU of ZIKV MR766); 912116 and 411359 (both infected with 10^4^ PFU of ZIKV French Polynesia isolated in 2013); 634675, 566628, and 311413 (all infected with 10^4^ PFU of ZIKV PRVABC59 isolated in Puerto Rico in 2015). **B)** Mean value of viral load status of ZIKV (strain PRVABC59) in all SIVtm-ZIKV co-infected animals (n = 4, blue) and in all ZIKV PRVABC59 infected animals (n = 3, red) in days post ZIKV inoculation. Bars indicate standard deviation (± SD) of mean values.
(TIF)

S2 Fig Body weight and temperature status of simian immunodeficiency virus and chimeric simian human immunodeficiency virus—Zika virus (SIV/SHIV-ZIKV) co-infected macaques versus macaques infected with ZIKV alone (from David O’Connor’s group). Rhesus macaques (n = 6) chronically infected with either SIVmac239 (n = 4) or SHIV3618MTF (n = 2) were also inoculated subcutaneously with 10^4^ plaque forming unit (PFU) of ZIKV PRVABC59.The body weight, and temperature records were performed according to the study plan on blood collection in S1 Fig. **A)** Body weight (Kg) records of individual animals on days pre and post inoculation with ZIKV. The reference numbers of the animals studied previously are also given in their labels (https://zika.labkey.com/project/OConnor/begin.view); 295022 (infected with 10^4^ PFU of ZIKV MR766); 912116 and 411359 (both infected with 10^4^ PFU of ZIKV French Polynesia isolated in 2013); 634675, 566628, and 311413 (all infected with 10^4^ PFU of ZIKV PRVABC59 isolated in Puerto Rico in 2015). **B)** Mean value of body weight (Kg) records of all SIV-ZIKV co-infected animals (n = 4, blue) and in all ZIKV PRVABC59 infected animals (n = 3, red) in days pre-and post ZIKV inoculation. Bars indicate standard deviation (±SD) of mean values. **C)** Body temperature (Celsius) records of individual animals on days pre-and post-inoculation with ZIKV. The reference number of the animals studied previously are also given in their labels https://zika.labkey.com/project/OConnor/begin.view; 295022 (infected with 10^4^ PFU of ZIKV MR766); 912116 and 411359 (both infected with 10^4^ PFU of ZIKV French Polynesia isolated in 2013); 634675, 566628, and 311413 (all infected with 10^4^ PFU of ZIKV PRVABC59 isolated in Puerto Rico in 2015). **D)** Mean value of body temperature (Celsius) records of all SIV-ZIKV co-infected animals (n = 4, blue) and in all ZIKV PRVABC59 infected animals (n = 3, red) in days pre-and post ZIKV inoculation. Bars indicate standard deviation (±SD) of mean values.
(TIF)

S1 Table Raw data of cytokines/chemokines measurement screening in simian immunodeficiency virus—Zika virus (SIV-ZIKV) co-infected monkeys. Rhesus macaques (n = 3) chronically infected with SIVmac239 were also inoculated subcutaneously with 10^4^ plaque forming unit (PFU) of ZIKV PRVABC59. The blood collections were performed according to the study plan on 0, 4, 7, 9, 15, 26 and 51 days post inoculation (DPI) with ZIKV. Day 0 (D0) was the day of inoculation with ZIKV. From collected serum samples on D0 to D26 post inoculation with ZIKV, Luminex assay was performed to screen all relevant cytokines/chemokines measurements. Using R, a heatmap was designed and drawn based on the median value (n = 3) of cytokines/chemokines measurements on D0, D4, D7, D9 and D26 post inoculation with ZIKV in SIV-ZIKV co-infected animals. **A)** Raw data of Luminex assay. **B)** Log2 of the raw data of which median values were calculated and used to draw the heat map **(Fig 2)** using R program software.
(DOCX)
