# Supplementary figures and images for: SIV/SHIV-Zika co-infection does not alter disease pathogenesis in adult non-pregnant rhesus macaque model
Source: PLoS Negl Trop Dis. 2018 Oct 25;12(10):e0006811. doi: 10.1371/journal.pntd.0006811 (PMC6201872; doi:10.1371/journal.pntd.0006811)

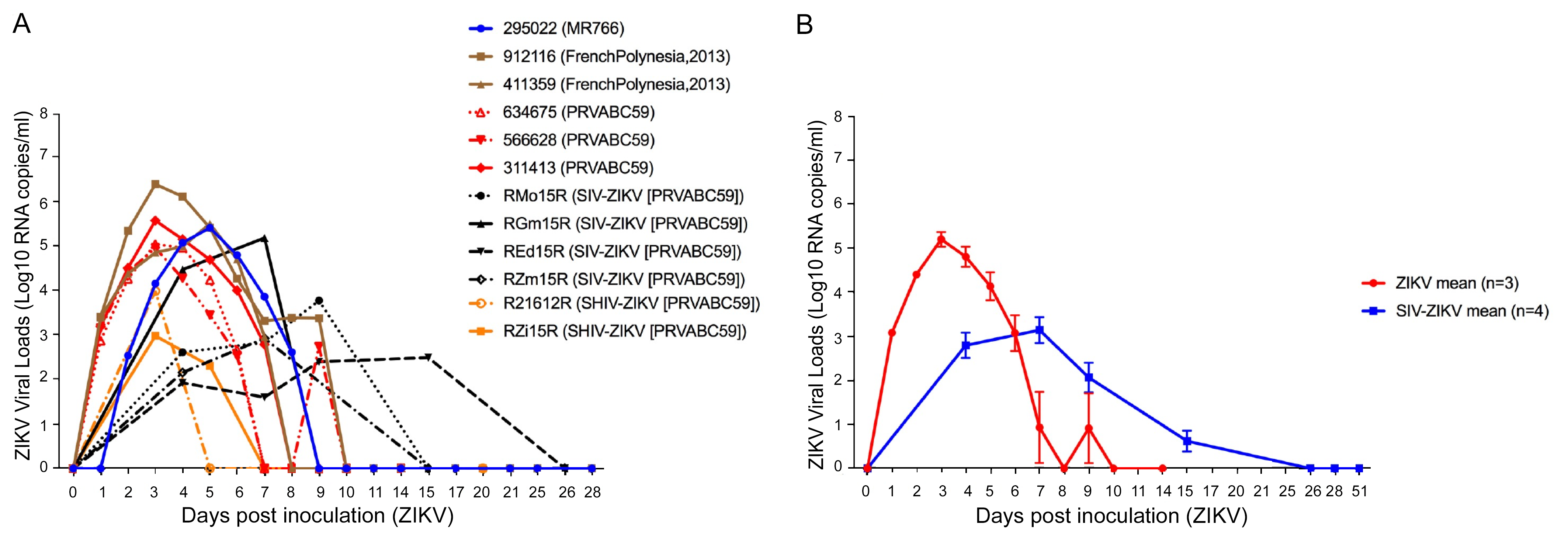

Supplement: S1 Fig — Rhesus macaques (RM; n = 6) chronically infected with either SIVmac239 (n = 4) or SHIV3618MTF (n = 2) were also inoculated subcutaneously with 104 plaque forming unit (PFU) of ZIKV PRVABC59. The blood collections were performed according to the study plan on 0, 4, 7, 9, 15, 26 and 51 days post inoculation (DPI) with ZIKV for SIV co-infected RM and on 0, 3, 5, 7, 10, and 20 DPI with ZIKV for SHIV co-infected RM. Day 0 (D0) was the day of inoculation with ZIKV. RNA was extracted from collected plasma samples using the QiAmp RNA mini kit (Qiagen, Valencia, CA), and viral loads were measured using one-step real time RT-PCR detection method targeting Gag gene of SIV. Viral loads were presented in Log10 RNA copies per milliliter (ml) of plasma. A) Viral load status of ZIKV in SIV (black, this study), SHIV (orange, this study)–ZIKV co-infected RM, in ZIKV PRVABC59 (red)—infected RM, in ZIKV FrenchPolynesia, 2013 (brown)—infected RM and in ZIKV MR766 (blue)—infected RM in days post ZIKV inoculation. The reference number for animals studied previously is also given in their labels (https://zika.labkey.com/project/OConnor/begin.view); 295022 (infected with 104 PFU of ZIKV MR766); 912116 and 411359 (both infected with 104 PFU of ZIKV French Polynesia isolated in 2013); 634675, 566628, and 311413 (all infected with 104 PFU of ZIKV PRVABC59 isolated in Puerto Rico in 2015). B) Mean value of viral load status of ZIKV (strain PRVABC59) in all SIVtm-ZIKV co-infected animals (n = 4, blue) and in all ZIKV PRVABC59 infected animals (n = 3, red) in days post ZIKV inoculation. Bars indicate standard deviation (± SD) of mean values. (TIF) [file pntd.0006811.s001.tif]

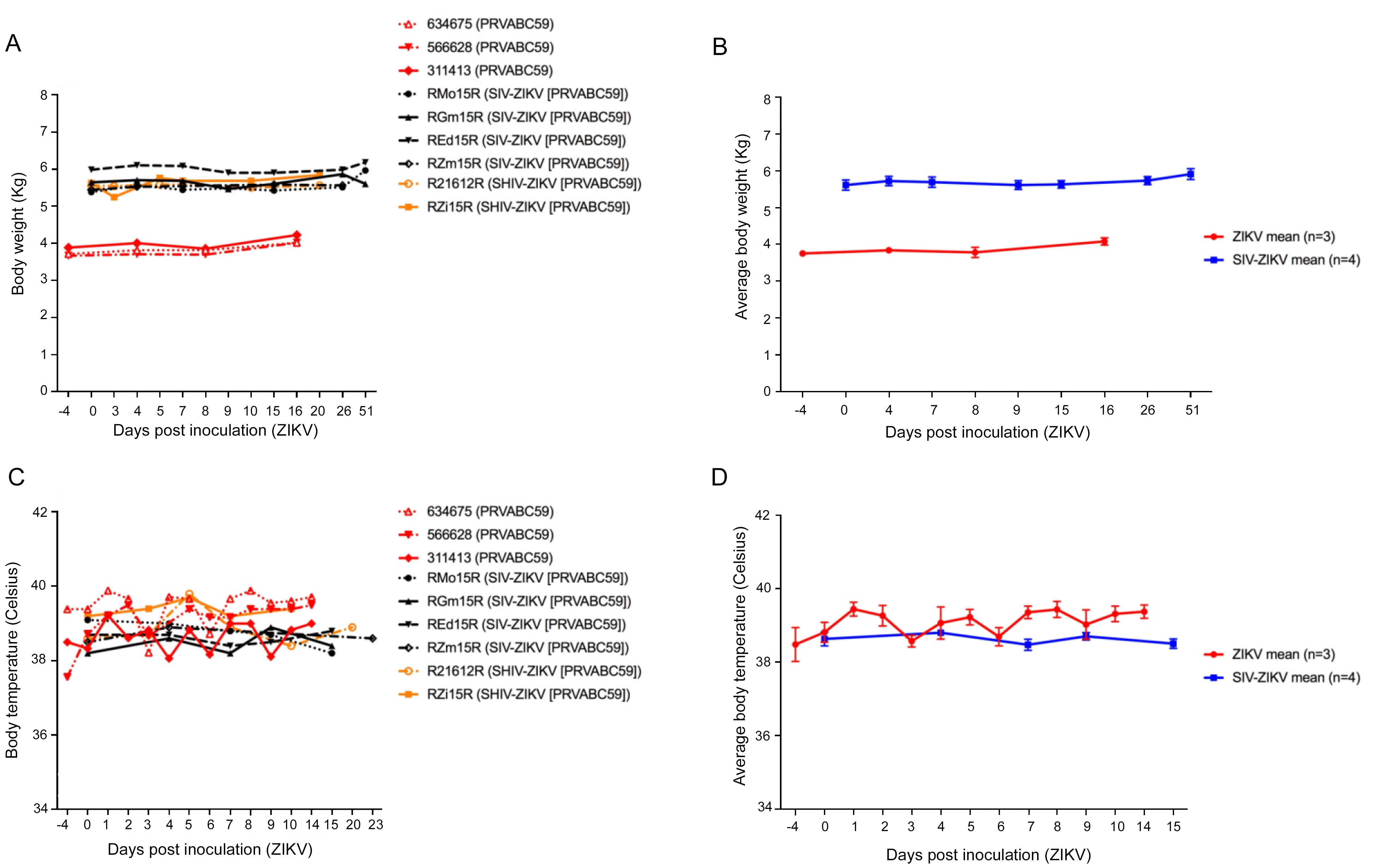

Supplement: S2 Fig — Rhesus macaques (n = 6) chronically infected with either SIVmac239 (n = 4) or SHIV3618MTF (n = 2) were also inoculated subcutaneously with 104 plaque forming unit (PFU) of ZIKV PRVABC59.The body weight, and temperature records were performed according to the study plan on blood collection in S1 Fig. A) Body weight (Kg) records of individual animals on days pre and post inoculation with ZIKV. The reference numbers of the animals studied previously are also given in their labels (https://zika.labkey.com/project/OConnor/begin.view); 295022 (infected with 104 PFU of ZIKV MR766); 912116 and 411359 (both infected with 104 PFU of ZIKV French Polynesia isolated in 2013); 634675, 566628, and 311413 (all infected with 104 PFU of ZIKV PRVABC59 isolated in Puerto Rico in 2015). B) Mean value of body weight (Kg) records of all SIV-ZIKV co-infected animals (n = 4, blue) and in all ZIKV PRVABC59 infected animals (n = 3, red) in days pre-and post ZIKV inoculation. Bars indicate standard deviation (±SD) of mean values. C) Body temperature (Celsius) records of individual animals on days pre-and post-inoculation with ZIKV. The reference number of the animals studied previously are also given in their labels https://zika.labkey.com/project/OConnor/begin.view; 295022 (infected with 104 PFU of ZIKV MR766); 912116 and 411359 (both infected with 104 PFU of ZIKV French Polynesia isolated in 2013); 634675, 566628, and 311413 (all infected with 104 PFU of ZIKV PRVABC59 isolated in Puerto Rico in 2015). D) Mean value of body temperature (Celsius) records of all SIV-ZIKV co-infected animals (n = 4, blue) and in all ZIKV PRVABC59 infected animals (n = 3, red) in days pre-and post ZIKV inoculation. Bars indicate standard deviation (±SD) of mean values. (TIF) [file pntd.0006811.s002.tif]
