# Supplementary material for: SIV/SHIV-Zika co-infection does not alter disease pathogenesis in adult non-pregnant rhesus macaque model
Source: PLoS Negl Trop Dis. 2018 Oct 25;12(10):e0006811. doi: 10.1371/journal.pntd.0006811 (PMC6201872; doi:10.1371/journal.pntd.0006811)
Supplement: S1 Table — Rhesus macaques (n = 3) chronically infected with SIVmac239 were also inoculated subcutaneously with 104 plaque forming unit (PFU) of ZIKV PRVABC59. The blood collections were performed according to the study plan on 0, 4, 7, 9, 15, 26 and 51 days post inoculation (DPI) with ZIKV. Day 0 (D0) was the day of inoculation with ZIKV. From collected serum samples on D0 to D26 post inoculation with ZIKV, Luminex assay was performed to screen all relevant cytokines/chemokines measurements. Using R, a heatmap was designed and drawn based on the median value (n = 3) of cytokines/chemokines measurements on D0, D4, D7, D9 and D26 post inoculation with ZIKV in SIV-ZIKV co-infected animals. A) Raw data of Luminex assay. B) Log2 of the raw data of which median values were calculated and used to draw the heat map (Fig 2) using R program software. (DOCX) [file pntd.0006811.s003.docx]

**S1 Table.**

**A.**

| **Luminex raw data** | RMo15R-D0 | REd15R-D0 | RGm15R-D0 | RMo15R-D4 | REd15R-D4 | RGm15R-D4 | RMo15R-D7 | REd15R-D7 | RGm15R-D7 | RMo15R-D9 | REd15R-D9 | RGm15R-D9 | RMo15R-D26 | REd15R-D26 | RGm15R-D26 |
| --- | --- | --- | --- | --- | --- | --- | --- | --- | --- | --- | --- | --- | --- | --- | --- |
| SDF-1a | 33 | 21 | 30 | 27 | 31 | 19 | 6 | 25 | 13 | 17 | 17 | 14 | 11 | 12 | 20 |
| IL-6 | 13 | 21 | 1 | 2 | 4 | 1 | 8 | 4 | 5 | 6 | 6 | 1 | 2 | 10 | 1 |
| BLC/CXCL13 | 846 | 3,740 | 1,116 | 3,571 | 19,662 | 1,011 | 416 | 19,662 | 1,562 | 1,230 | 10,261 | 994 | 2,348 | 18,537 | 5,243 |
| IP-10 | 207 | 248 | 159 | 217 | 151 | 251 | 260 | 162 | 349 | 134 | 159 | 302 | 167 | 209 | 304 |
| IL-1Ra | 588 | 853 | 283 | 325 | 420 | 288 | 651 | 531 | 739 | 336 | 419 | 349 | 266 | 497 | 316 |
| IL-7 | 4 | 5 | 11 | 0 | 1 | 3 | 0 | 2 | 4 | 1 | 2 | 3 | 0 | 2 | 3 |
| IL-8 | 4,115 | 9,726 | 3,124 | 461 | 4,498 | 1,153 | 528 | 2,835 | 876 | 1,416 | 5,187 | 1,101 | 975 | 3,422 | 1,166 |
| IFN-a | 154 | 10 | 1 | 79 | 16 | 11 | 150 | 20 | 71 | 93 | 13 | 9 | 81 | 34 | 21 |
| IFN-g | 5 | 24 | 1 | 4 | 7 | 1 | 7 | 7 | 1 | 7 | 8 | 1 | 4 | 11 | 1 |
| Eotaxin | 110 | 70 | 104 | 161 | 56 | 159 | 88 | 58 | 170 | 52 | 47 | 155 | 90 | 53 | 158 |
| RANTES | 1,750 | 2,881 | 892 | 275 | 1,989 | 3,982 | 447 | 2,669 | 1,916 | 762 | 2,313 | 2,383 | 422 | 1,131 | 1,333 |
| IL-1b | 6 | 0 | 0 | 2 | 0 | 3 | 5 | 0 | 3 | 5 | 0 | 2 | 2 | 0 | 1 |
| I-TAC | 58 | 50 | 22 | 37 | 25 | 31 | 35 | 45 | 45 | 34 | 52 | 49 | 29 | 34 | 37 |
| MCP-1 | 180 | 82 | 112 | 142 | 133 | 100 | 125 | 161 | 93 | 96 | 107 | 148 | 120 | 113 | 101 |
| GRO-a | 575 | 600 | 236 | 187 | 381 | 85 | 368 | 486 | 134 | 178 | 422 | 164 | 266 | 388 | 140 |
| MIF | 11,701 | 7,193 | 5,984 | 4,744 | 5,046 | 12,168 | 11,333 | 4,677 | 4,696 | 5,332 | 3,957 | 12,168 | 4,289 | 12,168 | 12,168 |

**B.**

| **Log2 of Luminex data** | RMo15R-D0 | REd15R-D0 | RGm15R-D0 | RMo15R-D4 | REd15R-D4 | RGm15R-D4 | RMo15R-D7 | REd15R-D7 | RGm15R-D7 | RMo15R-D9 | REd15R-D9 | RGm15R-D9 | RMo15R-D26 | REd15R-D26 | RGm15R-D26 |
| --- | --- | --- | --- | --- | --- | --- | --- | --- | --- | --- | --- | --- | --- | --- | --- |
| SDF-1a | 0.237039 | -0.41503 | 0.0995356 | -0.05246 | 0.146841 | -0.5594274 | -2.22239 | -0.16349 | -1.106915 | -0.71989 | -0.71989 | -1 | -1.34792 | -1.22239 | -0.48542 |
| IL-6 | 0.156119 | 0.847996 | -3.544320 | -2.54432 | -1.54432 | -3.5443205 | -0.54432 | -1.54432 | -1.222392 | -0.95935 | -0.95935 | -3.544320 | -2.54432 | -0.22239 | -3.54432 |
| BLC/CXCL13 | -1.16777 | 0.976532 | -0.768168 | 0.909822 | 3.370823 | -0.9107225 | -2.19185 | 3.370823 | -0.283111 | -0.62784 | 2.432593 | -0.935187 | 0.304926 | 3.285830 | 1.463887 |
| IP-10 | 0.016354 | 0.277063 | -0.364249 | 0.084418 | -0.43872 | 0.29441121 | 0.345235 | -0.33728 | 0.7699508 | -0.61104 | -0.36424 | 0.5612723 | -0.29342 | 0.030226 | 0.570795 |
| IL-1Ra | 0.033090 | 0.569820 | -1.021923 | -0.82228 | -0.45233 | -0.9966566 | 0.179932 | -0.11401 | 0.362849 | -0.77426 | -0.45577 | -0.719498 | -1.11129 | -0.20947 | -0.86280 |
| IL-7 | -0.73696 | -0.41503 | 0.7224660 | -0.73696 | -2.73696 | -1.1520031 | -0.73696 | -1.73696 | -0.736965 | -2.73696 | -1.73696 | -1.152003 | -5.92139 | -1.73696 | -1.15200 |
| IL-8 | -0.45863 | 0.782319 | -0.856132 | -3.61668 | -0.33024 | -2.2941345 | -3.42091 | -0.99617 | -2.690524 | -1.99770 | -0.12462 | -2.360712 | -2.53605 | -0.72468 | -2.27795 |
| IFN-a | 1.485426 | -2.45943 | -5.781359 | 0.522421 | -1.78135 | -2.3219281 | 1.447458 | -1.45943 | 0.3683874 | 0.757799 | -2.08092 | -2.611434 | 0.558490 | -0.69389 | -1.38904 |
| IFN-g | -0.98404 | 1.278991 | -3.883737 | -1.30597 | -0.49861 | -3.8837375 | -0.49861 | -0.49861 | -3.305970 | -0.49861 | -0.30597 | -3.883737 | -1.30597 | 0.153461 | -3.88373 |
| Eotaxin | 0.216575 | -0.43550 | 0.1356550 | 0.766132 | -0.75742 | 0.74809834 | -0.10535 | -0.70680 | 0.8446063 | -0.86434 | -1.01019 | 0.7113397 | -0.07293 | -0.83686 | 0.738996 |
| RANTES | -0.07313 | 0.646080 | -1.045374 | -2.74298 | 0.111553 | 1.11300359 | -2.04214 | 0.535809 | 0.0576079 | -1.27262 | 0.329275 | 0.3722893 | -2.12517 | -0.70289 | -0.46581 |
| IL-1b | 1.510000 | -3.71881 | -3.718818 | -0.07496 | -3.71881 | 0.51000044 | 1.246966 | -3.71881 | 0.5100004 | 1.246966 | -3.71881 | -0.074962 | -0.07496 | -3.71881 | -1.07496 |
| I-TAC | 0.420575 | 0.206450 | -0.977973 | -0.22795 | -0.79354 | -0.483209 | -0.30812 | 0.054447 | 0.0544477 | -0.34994 | 0.263034 | 0.1773045 | -0.57942 | -0.34994 | -0.22795 |
| MCP-1 | 0.529921 | -0.60437 | -0.154577 | 0.187815 | 0.093350 | -0.3180758 | 0.003852 | 0.368984 | -0.422773 | -0.37696 | -0.22046 | 0.2475214 | -0.05504 | -0.14175 | -0.30372 |
| GRO-a | 0.289878 | 0.351278 | -0.994896 | -1.33064 | -0.30389 | -2.4681488 | -0.35397 | 0.047272 | -1.811450 | -1.40180 | -0.15644 | -1.519987 | -0.82225 | -0.27762 | -1.74825 |
| MIF | 0.496723 | -0.20524 | -0.470725 | -0.80573 | -0.71669 | 0.55320895 | 0.450621 | -0.82625 | -0.820403 | -0.63715 | -1.06742 | 0.5532089 | -0.95119 | 0.553208 | 0.553208 |
